# Factors affecting reproductive performance in the Swedish Bernese mountain dog

**DOI:** 10.1186/s13028-022-00646-x

**Published:** 2022-10-21

**Authors:** Eva Axnér, Linda Sofia Rasmus, Toril Melangen

**Affiliations:** 1 Division of Reproduction, Department of Clinical Sciences, Faculty of Veterinary Medicine and Animal Science, Swedish University of Agricultural Sciences, 7054, 750 07 Uppsala, Sweden; 2 Björnmossestigen 9, 00890 Helsinki, Finland; 3 Fors 23 Fors Gård, 137 91 Västerhaninge, Sweden

**Keywords:** Caesarean section, Litter size, Seasonality, Puppy mortality

## Abstract

**Background:**

Good reproductive performance is fundamental for the development of a breed. Previous studies have indicated that the Bernese mountain dog has a relatively high prevalence of reproductive problems such as a high prevalence of dystocia and a low mean litter size. When reproduction is impaired, selection for other traits, including improved health, will become more difficult. The aim of this study was therefore to evaluate reproductive data and factors affecting these in the Bernese mountain dog. Data collected by the Swedish Sennenhund Club during the years 2010–2020 were evaluated by statistical analyses.

**Results:**

Information from 1287 reported matings were included with a total of 614 bitches and 399 sires. For five reported matings that did not result in a litter, there was no information about the male identity. The reported matings resulted in 798 litters (62% whelping rate) from 502 bitches and 314 males. Paternal and maternal age had a significant effect on whelping rate with a negative effect of increasing age (P < 0.01). Median litter size at birth (LSB) was 6.00 (range 0–14) and was significantly affected by both paternal (P = 0.021) and maternal age (P < 0.001). Parity affected litter size at birth with a lower litter size in 4 year old bitches giving birth to their first litter compared to bitches giving birth to their second to fourth litters. Stillbirth occurred in 51.6% of the litters with a total of 15.4% puppies being stillborn. Total puppy mortality, including stillbirth, was 19.1%. The only factor affecting stillbirth was LSB while both LSB and season affected the risk of having post-natal puppy loss in the litter. The total prevalence of caesarean sections (CS) was 33.0%. The risk of CS decreased significantly with increasing parity and increased with increasing age. The risk of CS was significantly higher for litters with 1–2 puppies compared with litters with 3–9 puppies. The coefficient of inbreeding (F) calculated on 5 generations had no effect on any of the outcomes.

**Conclusions:**

Parity and maternal age had opposite effects on reproductive outcomes with a positive effect of parity on increasing litter size and decreasing CS rate. The proportion of unsuccessful matings was high with a negative effect of increasing age of both males and females.

## Background

The Bernese mountain dog is a large muscular breed with strong bone structure, originating from Switzerland (Fedaration cynologique internationale, FCI standard No 45). Previous publications indicate that the Bernese mountain dog has a relatively high prevalence of reproductive problems. A high prevalence of dystocia and a small litter size compared to similar sized dogs has been reported [1, 2]. According to data from the Norwegian Kennel Club mean litter size at birth was 6.4 ± 0.3 (mean ± SE) in 137 litters during a period of two years, 2006–2007. This was lower than the overall mean value (6.9) for large sized dogs [2]. The Bernese mountain dog was ranked as the breed with the highest prevalence of pyometra in Swedish insurance data, with 66% developing the disease before the age of 10 years [3].

In addition to reproductive problems, the Bernese mountain dog has a relatively high prevalence of other types of morbidity and a short life expectancy compared with similar sized dogs [4, 5]. This might be related to a high level of inbreeding [4]. Inbreeding is also known to have a negative influence on reproduction [6].

A good reproductive performance is fundamental for the development of a breed. When reproduction is impaired, selection for other traits, including improved health, will become more difficult as breeding pairs selected to improve the breed may not produce the planned number of offspring. In order to improve reproductive success and to decrease the frequency of dystocia it is important to identify factors affecting these parameters. Previous studies on different breeds have shown that season of the year, age of the bitch, parity and inbreeding level may affect litter sizes in different breeds of dogs [2, 6–8]. Factors that may affect puppy survival at and after birth include dystocia, age of the mother and parity [9].

The Swedish Sennenhund Club has collected data about reproduction for several years. This study was initiated because of perceived problems with reproduction in the breed. The aim of the study was to identify factors that may influence litter size, unsuccessful matings, puppy mortality and the risk of caesarean section from data collected by the Swedish Sennenhund Club.

## Methods

### Study population

Data collected from breeders by the Swedish Sennenhund Dog Club, from the years 2010–2020 were included. There were 1287 observations from 614 bitches and 399 male dogs. The data were completed with registration data from the Swedish Kennel Club to get information about date of birth of the parents and parity of the bitch. Data about puppy mortality and dystocia are not included in the Swedish Kennel Club’s registration data, why litters that were not reported to the breed club are not included in the study except when specifically mentioned.

### Litter size and puppy losses

Litter size at birth (LSB) was calculated by adding the number of registered puppies with stillborn puppies and puppies that died after birth. Puppies that died after birth included euthanized puppies, as the cause of death was not always clear from the data files. Puppies that are euthanized usually have defects or are not viable for other reasons. The number of puppies remaining after puppy death was also registered by the breed club. However, for evaluations of litter size at registration (LSR) registration data from the Swedish Kennel Club was used.

### Caesarean sections

Birth by caesarean section (CS) was reported to the breed club and included in the evaluations as a binary outcome. For some litters, the breeder had commented that the bitch had received treatment for dystocia other than CS. However, other treatments than CS were not routinely reported why only dystocias resolved by a CS were included in the evaluations.

### Matings not resulting in a litter

Breeders reported planned matings to the breed club and if the bitch did not whelp after a mating. Month of expected birth was registered for litters not resulting in birth of a litter.

### Parental age

Parental age at birth was calculated as the time between birth of the parent and birth of the litter. For planned matings, not resulting in a litter, age was calculated from the first of the month the combination was expected to result in the birth of a litter. For 11 males, birth date was not available. Because of low number of observations in some ages, age was grouped into age classes. Because of a larger spread in the ages of males, ages of males were grouped into two more categories than ages of females.

### Parity

Parity was calculated from the number of litters registered in the Swedish Kennel Club before each litter. Data about litters in which all puppies died before registration was added to calculate parity, including litters without any surviving puppies. It was not possible to exclude that there might have been litters with 100% puppy mortality that were not reported to the breed club. Because of a low number of bitches having more than 4 litters (8 bitches), parity was categorised into 4 groups (parities 1 to 3, and parity ≥ 4). In the evaluation of litter size in 4 year old bitches, parity was categorised into 3 groups (parity 1, parity 2 and parity 3 + 4) because only one 4 year old bitch had parity 4.

### Season of birth of the litter

Month of birth, or expected month of birth for matings not resulting in a litter were grouped for season, winter (Dec–Feb), spring (Mar–May), summer (Jun–Aug), and autumn (Sep–Nov).

### Inbreeding coefficient

Coefficient of inbreeding of the litters was provided by the Swedish Sennenhund Club. The coefficient of inbreeding (F) is calculated on 5 generations by the tabular method [10] in the Swedish Kennel Club’s registry data. Information about inbreeding was missing in 94 observations.

### Statistics

Statistical calculations were made in R (R Core Team, 2021.09.0) [11]. Data about unsuccessful matings were removed from all models except for the model that evaluated this specific outcome. Dam and sire were included as random factors in all models. Multicollinearity was checked by evaluating correlation matrices of factors included in the models and by evaluating the variance inflation factor before including factors in the models.

To model LSB, a count variable, a generalised mixed model was used (glmer.nb). Parental age, and F were included as numeric factors. Parity and season were included as categorical factors. Before fitting the model, linearity assumption of predictors (age of dam and sire) were checked by plotting the numeric predictors against the outcome variable LSB. The model was tested with and without interactions between age of the dam and sire, and between parity and age of the dam. Interactions were removed from the final models as they were not significant. To further evaluate the effect of parity on LSB, an evaluation with only 4-year old bitches was made, as parity and age of the bitch seemed to have opposite effects. Overdispersion was detected by evaluating the model’s deviance and was accounted for by using a negative binomial distribution that can estimate a variance that deviates from the mean. Categorical factors that were significant were further evaluated with Tukey’s pairwise comparisons.

To model the outcomes; CS, stillbirth in the litter, postnatal puppy mortality in the litter, and unsuccessful matings, a binomial generalised mixed model was used (glmer, family binomial, glmerControl, optimizer = “bobyqa”). Age of the dam, parity, and season were included as categorical factors. For evaluation of stillbirth and postnatal puppy mortality, CS was used as a fixed binomial variable. Stillbirth in the litter was also included in the model as a fixed binomial variable for the outcome or postnatal puppy mortality in the litter. The coefficient of inbreeding was used as a numeric factor in all models. Litter sizes were categorized into five categories (1–2, 3–4, 5–7, 8–9 and ≥ 10 puppies). Paternal age was not included in evaluations of factors occurring after fertilisation (CS, stillbirth, and postnatal puppy mortality), as it was considered unlikely to have an effect. Odd ratios (OR) were calculated for categorical factors in the binomial models. The risk of CS in the second parity compared with the first in the same bitch was evaluated with a chi-squared test.

Anova tables were constructed from the models to evaluate overall P-values of factors. Because of a large number of missing values for F, calculations were repeated without this factor. Results are reported without F included unless stated otherwise. P ≤ 0.05 were considered significant.

### Ethical statement

Data from the Swedish Kennel Clubs registry are from an open database. Breeders are informed that data subjected to the Swedish Sennenhund Club health registers can be used to evaluate the health status in the breed. Data collected from the Swedish Sennenhund Club have been handled in accordance with regulations for processing data at the Swedish University of Agricultural Sciences.

## Results

### Study population

A total of 502 bitches gave birth to one to four litters and 314 males sired one to 17 litters included in the study (Table 1). Ages of females varied between 22.6 and 90.9 months and of sires between 14.2 and 131.8 months. One female that was only five months old and that did not give birth to a litter was excluded. It is not allowed to mate bitches younger than 18 months of age in Sweden and it was unclear if this was an intentional mating. Parity ranged between 1 and 5. A comparison with litters registered in the Swedish Kennel Club during the same period showed that 85.6% (798/932) of all registered litters and 81.1% (3889/4795) of all registered puppies were included in our data. Data on the number of litters that were born, number of matings not resulting in a litter, and the number of CS are shown in Table 1.

**Table 1:** Descriptive data on the study populations

	Puppy level (% of total)	Litter level median (range) Proportion (number of events/number of litters)
Number of matings	1287	–
Number of whelpings	798 (62.0)	–
Caesarean sections (of whelpings)	–	33.0 (263/798)
Number of born puppies	4815	6.0 (1.0–14.0)
Stillborn/Litters with stillbirth	740 (15.4)	51.6 (412/798)
Died after birth/Litters with postnatal puppy mortality	179 (3.7)	16.0 (128/798)
Total puppy losses/Litters with puppy losses	919 (19.1)	59.6 (476/798)
Registered puppies^a^	3889 (80.8)	5.0 (0–13)

The number of puppies at weaning differed slightly between the kennel clubs registry and the breed club’s data for the included litters (3889 vs. 3896 puppies)

^a^The number of registered puppies are from the Swedish kennel clubs registry

### Puppy mortality

Stillbirth occurred in 412 litters (51.6% of litters, 1–9 puppies/litter) and in 128 litters, puppies died after birth (1–6 puppies/litter) (Table 1). Stillbirth and/or puppies that died between birth and registration (total puppy mortality) occurred in 476 litters (59.6%). All puppies were lost in 34 litters (1–9 puppies). Data of puppy mortality and mortality at litter level are shown in Tables 1 and 2. The only factor affecting stillbirth was LSB while both LSB and season affected the risk of having post-natal puppy loss in the litter (Table 2).

**Table 2:** Puppy mortality in litters

		Outcome							
		Stillbirth			Mortality of live-born puppies				
Factor	Factor level	Proportion % (n at factor level)	OR (95% CI)	P-value	Proportion % (n at factor level)		OR (95% CI)		P-value
Age of bitch	Age 1–2	57.0 (242)	Reference	0.81^a^	15.7 (242)	Reference		0.87^a^	
	Age 3	51.5 (237)	0.78 (0.51–1.20)	–	17.3 (237)	1.05 ( 0.59–1.85)		–	
	Age 4	48.8 (168)	0.76 (0.45–1.28)	–	17.9 (168)	1.28 (0.63–2.59)		–	
	Age 5	48.0 (100)	0.73 (0.36–1.45)	–	13.0 (100)	0.93 ( 0.36–2.40)		–	
	Age ≥ 6	43.1 (51)	0.77 (0.30–1.94)	–	11.8 (51)	0.73 (0.19–2.85)		–	
Parity	Parity1	53.1 (426)	Reference	0.83^a^	16.2 (426)	Reference		0.48^a^	
	Parity 2	52.5 (236)	1.16 (0.76–1.79)	–	18.6 (236)	1.16 (0.66–2.04)		–	
	Parity 3	46.9 (98)	0.97 (0.50–1.84)	–	10.2 (98)	0.61 (0.24–1.56)		–	
	Parity ≥ 4	42.1 (38)	0.91 (0.34–2.47)	–	13.2 (38)	1.00 (0.24–4.18)		–	
Season	Winter	48.9 (174)	Reference	0.052^a^	9.2 (174)	Reference		Reference	
	Spring	55.3 (291)	1.26 (0.83–1.92)	–	16.8 (291)	2.06 (1.09–3.91)		0.026	
	Summer	56.2 (178)	1.41 (0.88–2.24)	–	20.2 (178)	2.72 (1.37–5.40)		0.0041	
	Autumn	42.6 (155)	0.76 (0.47–1.24)	–	17.4 (155)	2.15 (1.06–4.37)		0.034	
LSB	LSB 1–2	35.5 (107)	Reference	Reference	9.3 (107)	Reference		Reference	
	LSB 3–4	39.9 (163)	1.19 (0.69–2.06)	0.52	11.7 (163)	1.41 (0.59–3.34)		0.44	
	LSB 5–7	49.0 (257)	1.75 (1.05–2.90)	0.031	21.9 (257)	3.25 (1.46–7.20)		0.0037	
	LSB 8–9	66.3 (172)	3.62 (2.07–6.33)	< 0.001	13.4 (172)	1.83 (0.76–4.42)		0.18	
	LSB ≥ 10	69.7 (99)	4.28 (2.62–8.10)	< 0.001	20.2 (99)	3.00 (1.21–7.45)		0.018	
CS	CS	49.4 (263)	0.96 (0.68–1.34)	0.82	18.6 (263)	1.49 (0.95–2.33)		0.082	
Stillbirth	Stillbirth	–	–	–	15.5 (412)	0.80 (0.52–1.24)		0.32	

^a^Factors that were not significant in in the general ANOVA were not further evaluated with comparisons at factor level

### Effect of parental age and parity on litter size at birth, and the risk of unsuccessful mating

The median LSB was 6.0 (interquartile range 4–8). Only 16 observations (1.2%) were from bitches that were 7 years old at the time of expected whelping (of which 7 did not produce a litter) and no bitch was older than 7 years, while 97 (7.6%) of the observed males with known age were ≥ 7 years. Age of both the dam (P < 0.001) and the sire (P = 0.021) significantly affected LSB (Table 3). Of all included litters, a majority, 426 (53.8%) were born in the first parity. The mean age at the first parity was 36.56 months with 233/426 (54.7%) of first-parity bitches being < 3 years of age. Parity did not affect LSB in the complete dataset (P = 0.14), but had a positive effect on litter size in the evaluation of only 4 year old bitches with a significantly lower mean LSB in parity one (Tables 3 and 4). Age of both the bitch and the male, as well as parity, significantly affected the risk of an unsuccessful mating with an increased risk as age increased (Table 3).

**Table 3:** LSB and unsuccessful matings in relation to age of the bitch at whelping or mating

		Outcome				
		LSB		Unsuccessful matings		
Factor	Factor level	Mean ± SD (n)	P-value	Proportion (n)	OR (95% CI) SE*	P-value
Bitch age	1–2	6.4 ± 2.9 (242)	< 0.001^a^	33.9 (366)	0.037	< 0.001^a^
	3	6.2 ± 3.0 (237)	–	36.8( 375)	–	–
	4	5.8 ± 2.9 (168)	–	36.4 (264)	–	–
	5	5.7 ± 3.1 (100)	–	45.36 (183)	–	–
	≥ 6	5.0 ± 2.3 (51)	–	48.5 (99)	–	–
Male age	1	6.1 ± 3.0 (106)	0.021^a^	30.1 (156)	0.075	< 0.001^a^
	2	6.2 ± 2.8 (176)	–	33.8( 284)	–	–
	3	6.4 ± 2.9 (163)	–	37.8 (254)	–	–
	4	6.0 ± 3.2 (139)	–	36.4 (206)	–	–
	5	5.7 ± 2.7 (89)	–	42.5 (160)	–	–
	6	5.8 ± 2.9 (73)	–	42.0(119)	–	–
	≥ 7	5.2 ± 2.8 (50)		49.5 (97)	–	–
Parity	1	6.1 ± 3.0 (426)	0.14^b^	39.4 (704)	Reference	Reference
	2	6.1 ± 3.0 (236)	–	33.0 (349)	0.53 (0.37–0.75)	< 0.001
	3	5.8 ± 2.7 (98)	–	43.4 (175)	0.61 (0.37–1.02)	0.058
	≥ 4	5.4 ± 2.7 (38)	–	33.3 (57)	0.35 (0.17–0.78)	0.010
Season	Winter	6.0 ± 3.1 (174)	0.23^b^	34.1 (264)	Reference	0.50^a^
	Spring	6.3 ± 3.0 (291)	–	37.6 (465)	1.13 (0.80–1.60)	–
	Summer	5.9 ± 2.8 (178)	–	38.7 (292)	1.15 (0.79–1.69)	–
	Autumn	5.8 ± 2.8 (155)	–	41.9 (265)	1.35 (0.92–2.00)	–

Means ± SDs and proportions shown in the table are from the study population

^a^Bitch and male age were included as numeric factors only

^b^Factors that were not significant in in the general ANOVA were not further evaluated at factor level

**Table 4:** Litter size at birth (LSB) in 4-year old bitches. n = number of observations

Factor	Factor level	Means ± SD	P-value
Parity	Parity 1, n = 48	4.7 ± 3.0^a^	Reference
	Parity 2, n = 83	6.2 ± 2.8^b^	0.0025
	Parity 3–4, n = 37	6.3 ± 2.9^b^	0.0056
Season	Winter, n = 47	6.0 ± 3.0	0.41^c^
	Spring, n = 64	5.6 ± 3.0	–
	Summer, n = 30	5.3 ± 2.8	–
	Autumn, n = 27	6.3 ± 2.9	–
Male age^*^	Male age, n = 168	–	0.40

Means ± SDs shown in the table are from the study population

^ab^Rows with different letters also differs significantly in Tukey’s pairwise comparisons (P < 0.05)

^*^Mage age category was included as a numeric factor

^c^Factors that were not significant in in the general ANOVA were not further evaluated with comparisons at factor level

### Caesarean sections

Litters with 1–2 puppies made up 13.4% (107/798) of all litters and had a significantly higher risk of CS than litters with 3–9 puppies (Table 5). Bitches 1–2 years of age, had a lower risk of CS than older bitches. Parity had an opposite effect than age, with a decreased risk with increasing parity.

**Table 5:** The risk of caesarean sections (CS) in relation to age of the mother (years), parity, litter size at birth and (LSB)

Factor	Factor level	CS Rate % (n)	OR (95% CI interval)	P-value
Bitch age (years)	Age 1–2	28.9 (242)	Reference	Reference
	Age 3	35.4 (237)	1.79 (1.14–2.81)	0.011
	Age 4	33.9 (168)	2.22 (1.27–3.88)	0.0050
	Age 5	35.0 (100)	3.25 (1.58–6.71)	0.0014
	Age ≥ 6	33.3 (51)	4.82 (1.80–12.86)	0.0017
Parity	Parity 1	36.2 (426)	Reference	Reference
	Parity 2	31.4 (236)	0.49 (0.31–0.77)	0.0019
	Parity 3	27.6 (98)	0.30 (0.15–0.59)	< 0.001
	Parity ≥ 4	21.1 (38)	0.15 (0.05–0.46)	< 0.0011
LSB	LSB 1–2	50.5 (107)	Reference	Reference
	LSB 3–4	35.6 (163)	0.49 (0.28–0.85)	0.011
	LSB 5–7	26.8 (257)	0.36 (0.21–0.60)	< 0.001
	LSB 8–9	26.7 (172)	0.35 (.020–0.61)	< 0.001
	LSB ≥ 10	36.4 (99)	0.57 (0.31–1.06)	0.077
Season^a^	Winter	32.2 (174)	Reference	0.90
	Spring	32.6 (291)	1.04 (0.67–1.62)	–
	Summer	32.6 (178)	0.97 (0.59–1.59)	–
	Autumn	34.8 (155)	1.17 (0.70–1.93)	–
Stillbirth in the litter	Stillbirth	31.6 (412)	0.97 (0.70–1.36)	0.88

Proportions shown in the table are from the study population

^a^Factors that were not significant in in the general ANOVA were not further evaluated with comparisons at factor level

There were data about both first and second parity for 187 bitches of which 51 had a CS in their first parity (27.3%). Of these 51, 17 (33.3%) also had a CS in their second parity. Thirty-nine out of 136 (28.7%) bitches that did not have a CS in their first parity gave birth by CS in their second parity. This difference was not significant (P = 0.54).

### Effect of season

**Fig. 1:**
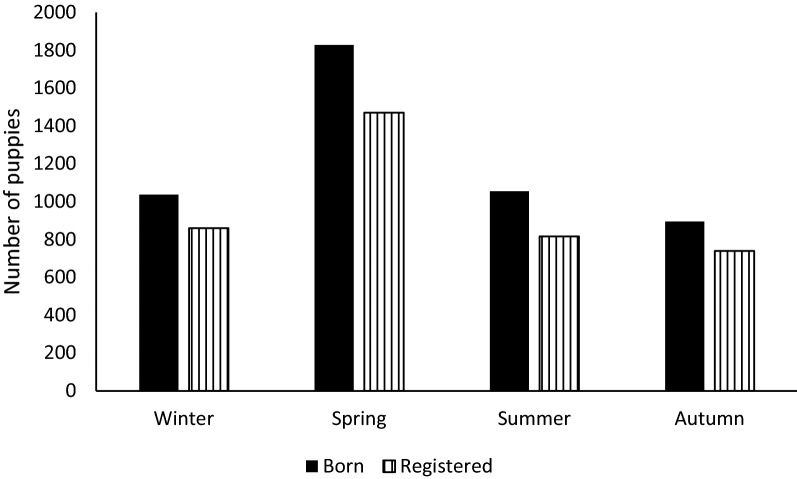
Number of puppies at birth and registration in different seasons

The highest number of litters and puppies were born during spring (Tables 2, 3, and 5, and Fig. 1). Season had no significant effect on any of the outcomes (LSB, CS, stillbirth, or unsuccessful matings) except the mortality risk of live born puppies (Table 2). Significantly fewer litters had post-natal puppy mortality when whelping was planned to occur during winter compared to all other seasons.

### Inbreeding coefficient

The mean inbreeding coefficient (F) for five generations was 0.97 (SD 1.17) with a range between 0 and 6.6. There were no effects of F on any of the reproductive outcomes.

## Discussion

Our data confirmed that mean litter size at birth was lower than expected compared with breeds of similar size as the Bernese mountain dog, the proportion of unsuccessful matings was high, and that puppy mortality, especially stillbirth, was in the higher range for the species [9, 12–14].

Parental age significantly influenced several of the outcomes in this study. Paternal and maternal age affected both the LSB and the risk of unsuccessful mating. At a paternal age of 7 years only approximately 50% of the planned matings resulted in a litter. A decrease in fertility in males might be related to semen quality, as well as mating problems. According to the first author’s experience, it is not uncommon for male dogs ≥ 7 years to have impaired semen quality. A negative correlation between age and the percentage of morphologically normal spermatozoa has been reported previously [15]. Aged dogs are also more likely to have testicular tumours that may have a negative effect on semen quality, even when clinically undetectable [16]. A decrease of litter size with advancing age has been shown previously [2, 7]. Interestingly, opposite to age, increasing parity had a positive effect on LSB with a lower mean litter size for first parity bitches at the age of 4 years compared with parities 2–4 at the same age. The risk of CS increased significantly with age while increasing parity decreased the risk. An increased risk of CS with age has been observed in other breeds [17]. Our data thus demonstrates opposite effects of parity and age in the Bernese mountain dog. In Norwegian Kennel Club data a more pronounced decrease with age in litter size was found for bitches of larger breeds compared with smaller, which is consistent with our results with a continuous decrease in litter size with increasing maternal age in the Bernese mountain dog [2]. Uterine health might be a contributing reason for declining fertility. With advanced age, bitches tend to develop cystic endometrial hyperplasia, which might interfere with fertility. The Bernese mountain dog has a high frequency of pyometra, a disease with higher frequency with increasing age indicating that uterine health is a problem in this breed [3, 18]. It is not allowed to mate bitches < 18 months of age according to Swedish animal welfare legislation. The Swedish Sennenhund Club currently recommends that a bitch should be at least two years old when she gives birth to her first litter to allow enough time of mental and skeletal maturation before breeding. From the results of this study it can thus be recommended to breed a bitch of this breed for the first time after 2 years of age but before 3 years of age. Unfortunately optimal age for reproductive performance might conflict with a desire to allow enough time for a breeding animal to undergo health tests and to get merits before breeding.

Similar to our results, Cornelius et al. [17] found that the highest dystocia rate was in small litters, and the lowest was found in litters of medium size (5–9). The risk of dystocia decreased with increasing parity in our study. It cannot be excluded that the decreasing risk with increasing parity was caused by factors other than the parity as such. For example, breeders might prefer to continue breeding from bitches with previous uncomplicated births. Unexpectedly, CS in the first parity did not seem to increase the risk in the second. However, only dystocias treated by CS were included in our evaluations and the causes of dystocia were not included. For example, a dystocia caused by fetal factors, such as malposition, might not increase the risk of repeated problems while dystocia caused by maternal factors are more likely to increase the risk for repeated dystocia. Therefore, we cannot conclude that repeated breeding from a bitch giving birth by CS is without risks for repeated problems in general. According to Swedish animal welfare regulations, it is not allowed to breed from a bitch that has given birth twice by CS. This was also the maximum number of CS for any of the bitches included in this study.

Considering the results of a larger mean LSB in multiparous compared with primiparous bitches at 4-years of age together with a decreased risk of CS with increasing parity it might be concluded that pregnancy have a positive effect on factors affecting future fertility.

The high proportion of planned matings that did not result in a whelping and a high frequency of litters with only 1–2 puppies might indicate improper timing of mating in some cases. Proper timing is especially important if semen quality is impaired. There was no consistent information about methods used for timing of mating or if progesterone measurements were performed in the data.

A total puppy mortality of 19.1% before registration is relatively high compared with the general dog population [9]. The majority of puppy losses was caused by stillbirth (15.4% of all puppies). The only factor that was significantly related with stillbirth in the litter was LSB with an increasing risk concomitantly to litter size. The mortality risk of live-born puppies in the litter was also significantly affected by litter size at birth. Surprisingly, CS did not affect puppy mortality. In other studies, dystocia has been shown to be a risk factor for stillbirth [17]. This difference might be because our data only considered dystocia treated by CS. It is possible that dystocia treated by prompt intervention by CS results in a shorter parturition time and better puppy survival compared with a prolonged course of medical treatments. It is, however, likely that in some of the included cases, medical treatment had been tried and failed before a decision of CS was made. To further elucidate factors contributing to stillbirth in the Swedish Bernese mountain dog a more detailed data collection including all cases of dystocia and prolonged birth process would be beneficial.

Although the domestic dog is considered non-seasonal, traces of seasonality has been found in some breeds with differences in litter size between seasons [6, 7]. In our study season only significantly affected the risk of postnatal puppy mortality. Opposite to the results by Schrack et al. [6] puppy mortality was lowest in the winter while they reported highest total puppy mortality in the winter. However, Schrack et al. [6] used the mean numbers of total puppy mortality in the litter and had highest litter sizes in the winter while our study evaluated the risk of puppy mortality in the litter as a binary outcome. Because puppy mortality is significantly higher in larger litters, differences between our studies might be attributed to an expected larger number of puppies dying in larger litters. Similar to the study by Schrack et al. [6], the largest number of litters and puppies were born during spring.

The inbreeding coefficient of the litter had no significant effect on any of the outcomes. There were, however, many missing values for this factor which might have affected the results. The true level of inbreeding may be underestimated when calculated on 5 generations compared with complete pedigrees or direct genotype-based methods [4]. When inbreeding was based on genotype, the average inbreeding in the Bernese mountain dog was 0.317, which is much higher than the values in this study [4]. The Bernese mountain dog have signs that can be interpreted as typical of inbreeding depression, such as reduced longevity, impaired reproduction and a high level of morbidity [5, 8]. It is a large breed and as such expected to have a reduced longevity and a higher morbidity rate compared with smaller sized breeds, but even compared to similar sized dogs longevity, reproduction and health seems to be impaired [2, 5].

The results of this study were based on data reported by the breeders. Therefore it is not possible to fully control the accuracy of the data. However, collection of such a large quantity of data would not be possible without the client based reports. Although, owner reported data has some limitations, the large quantity of data that is possible to collect with the help of owners is a strength.

## Conclusions

Litter sizes and the risk of unsuccessful matings were significantly negatively affected by maternal and paternal age. Age and parity of the bitch had opposite effects on LSB and the risk of CS with a positive effect of parity and a negative effect of age. Litters with 1–2 puppies were at the highest risk of CS. To improve reproductive outcomes it is recommended to breed bitches for the first time before the age of 3 years. It is also recommended to continue to register reproduction data in the breed, and to include more detailed data about dystocias in addition to the occurrence of CS. The study also demonstrates the importance of data collection by kennel, and breed clubs, to be able to identify factors that have a potential to improve health and reproduction in a breed.

## Data Availability

Datasets modified so that individual animals cannot be identified, analyzed during the current study are available from the corresponding author on reasonable request.

## Abbreviations

LSB: Litter size at birth
CS: Caesarean section
F: Inbreeding coefficient

## References

[1] Bergström A, Nodtvedt A, Lagerstedt AS, Egenvall A (2006). Incidence and breed predilection for dystocia and risk factors for cesarean section in a Swedish population of insured dogs. Vet Surg.

[2] Borge KS, Tønnessen R, Nødtvedt A, Indrebø A (2011). Litter size at birth in purebred dogs—a retrospective study of 224 breeds. Theriogenology.

[3] Jitpean S, Hagman R, Strom Holst B, Hoglund OV, Pettersson A, Egenvall A (2012). Breed variations in the incidence of pyometra and mammary tumours in Swedish dogs. Reprod Domest Anim.

[4] Bannasch D, Famula T, Donner J, Anderson H, Honkanen L, Batcher K (2021). The effect of inbreeding, body size and morphology on health in dog breeds. Canine Med Genet.

[5] Klopfenstein M, Howard J, Rossetti M, Geissbühler U (2016). Life expectancy and causes of death in Bernese mountain dogs in Switzerland. BMC Vet Res.

[6] Schrack J, Dolf G, Reichler IM, Schelling C (2017). Factors influencing litter size and puppy losses in the Entlebucher mountain dog. Theriogenology.

[7] Gavrilovic BB, Andersson K, Linde FC (2008). Reproductive patterns in the domestic dog—a retrospective study of the Drever breed. Theriogenology.

[8] Mandigers PJ, Ubbink GJ, Vanden Broek J, Bouw J (1994). Relationship between litter size and other reproductive traits in the Dutch kookier dog. Vet Q.

[9] Tønnessen R, Borge KS, Nødtvedt A, Indrebø A (2012). Canine perinatal mortality: a cohort study of 224 breeds. Theriogenology.

[10] Chang HL, Fernando RL, Grossman M (1991). On the principle underlying the tabular method to compute coancestry. Theor Appl Genet.

[11] R Core Team. R: a language and environment for statistical computing (2021.09.0). R foundation for statistical computing, Vienna, Austria. 2022. https://www.R-project.org/.

[12] Mugnier A, Mila H, Guiraud F, Brevaux J, Lecarpentier M, Martinez C (2019). Birth weight as a risk factor for neonatal mortality: breed-specific approach to identify at-risk puppies. Prev Vet Med.

[13] Indrebø A, Trangerud C, Moe L (2007). Canine neonatal mortality in four large breeds. Acta Vet Scand.

[14] Chastant-Maillard S, Guillemot C, Feugier A, Mariani C, Grellet A, Mila H (2017). Reproductive performance and pre-weaning mortality: preliminary analysis of 27,221 purebred female dogs and 204,537 puppies in france. Reprod Domest Anim.

[15] Rijsselaere T, Maes D, Hoflack G, de Kruif A, Van Soom A (2007). Effect of body weight, age and breeding history on canine sperm quality parameters measured by the hamilton-thorne analyser. Reprod Dom Anim.

[16] Peters MA, de Rooij DG, Teerds KJ, van de Gaag I, van Sluijs FJ (2001). Spermatogenesis and testicular tumours in ageing dogs. J Reprod Fertil Suppl.

[17] Cornelius AJ, Moxon R, Russenberger J, Havlena B, Cheong SH (2019). Identifying risk factors for canine dystocia and stillbirths. Theriogenology.

[18] Moxon R, Whiteside H, England GC (2016). Prevalence of ultrasound-determined cystic endometrial hyperplasia and the relationship with age in dogs. Theriogenology.

